# Dysregulation of the Bmi-1/p16^Ink4a^ pathway provokes an aging-associated decline of submandibular gland function

**DOI:** 10.1111/acel.12337

**Published:** 2015-03-31

**Authors:** Kimi Yamakoshi, Satoshi Katano, Mayu Iida, Hiromi Kimura, Atsushi Okuma, Madoka Ikemoto-Uezumi, Naoko Ohtani, Eiji Hara, Mitsuo Maruyama

**Affiliations:** 1 Department of Mechanism of Aging, Research Institute, National Center for Geriatrics and GerontologyObu, Aichi, 474-8511, Japan; 2 Division of Cancer Biology, The Cancer Institute, Japanese Foundation for Cancer ResearchKoto-ku, Tokyo, 135-8550, Japan; 3 Department of Regenerative Medicine, Research Institute, National Center for Geriatrics and GerontologyObu, Aichi, 474-8511, Japan; 4 Department of Applied Biological Science, Faculty of Science and Technology, Tokyo University of ScienceNoda, Chiba, 278-8510, Japan

**Keywords:** aging, Bmi-1, homeostasis, p16^Ink4a^, stem/progenitor cells, submandibular gland

## Abstract

Bmi-1 prevents stem cell aging, at least partly, by blocking expression of the cyclin-dependent kinase inhibitor p16^Ink4a^. Therefore, dysregulation of the Bmi-1/p16^Ink4a^ pathway is considered key to the loss of tissue homeostasis and development of associated degenerative diseases during aging. However, because Bmi-1 knockout (KO) mice die within 20 weeks after birth, it is difficult to determine exactly where and when dysregulation of the Bmi-1/p16^Ink4a^ pathway occurs during aging *in vivo*. Using real-time *in vivo* imaging of p16^Ink4a^ expression in Bmi-1-KO mice, we uncovered a novel function of the Bmi-1/p16^Ink4a^ pathway in controlling homeostasis of the submandibular glands (SMGs), which secrete saliva into the oral cavity. This pathway is dysregulated during aging *in vivo*, leading to induction of p16^Ink4a^ expression and subsequent declined SMG function. These findings will advance our understanding of the molecular mechanisms underlying the aging-related decline of SMG function and associated salivary gland hypofunction, which is particularly problematic among the elderly.

## Introduction

In higher eukaryotes, maintenance of adult stem and progenitor cells is indispensable for tissue homeostasis throughout the lifespan of the organism (Cheung & Rando, 2013). However, regulation of these processes declines with age, resulting in an increased incidence of various aging-associated degenerative diseases (Sharpless & DePinho, 2007; Liu & Rando, 2011; Behrens *et al*., 2014). Bmi-1 belongs to the PRC1, which is recruited to a locus due to the PRC2 which trimethylate H3K27, priming the Bmi-1-containing PRC1L ubiquitin ligase complex to silence a locus (Hernandez-Munoz *et al*., 2005; Bracken *et al*., 2007; Kotake *et al*., 2007) and is essential for self-renewal of several types of adult stem cells and/or proliferation of certain types of differentiated cells, such as pancreatic β cells (Lessard & Sauvageau, 2003; Molofsky *et al*., 2003, 2005; Park *et al*., 2003; Iwama *et al*., 2004; Dhawan *et al*., 2009; Biehs *et al*., 2013). For example, although knockout (KO) mice lacking Bmi-1 are born with normal numbers of stem cells, Bmi-1-KO mice exhibit postnatal self-renewal defects that lead to premature depletion of adult stem cells, which resembles accelerated aging (van der Lugt *et al*., 1994; Lessard & Sauvageau, 2003; Molofsky *et al*., 2003; Park *et al*., 2003; Robson *et al*., 2011). Therefore, the aging-associated decline of Bmi-1 function may lead to failure of adult stem cell homeostasis and subsequent aging-associated disruption of tissue repair mechanisms and subsequent onset of degenerative diseases. Thus, better understanding of the downstream mediators of the Bmi-1 pathway will likely facilitate the development of new strategies for prevention or intervention of aging-associated degenerative diseases.

Several downstream targets of the Bmi-1 pathway have been proposed (van der Lugt *et al*., 1996; Jacobs *et al*., 1999; Oguro *et al*., 2010). Among them, *p16*^*Ink4a*^ may be the strongest candidate for stem cell regulation (Jacobs *et al*., 1999; Molofsky *et al*., 2003, 2005; Park *et al*., 2003; Dhawan *et al*., 2009; Biehs *et al*., 2013). The cyclin-dependent kinase (CDK) inhibitor encoded by *p16*^*Ink4a*^ slows down or blocks cell cycle progression by preventing phosphorylation and inactivation of the retinoblastoma tumor suppressor protein (pRb) (Serrano *et al*., 1993; Hara *et al*., 1996). Moreover, *p16*^*Ink4a*^ expression levels dramatically increase in several tissues with age (Zindy *et al*., 1997; Krishnamurthy *et al*., 2004; Yamakoshi *et al*., 2009), coinciding with the onset of the aging-associated functional decline of adult stem or progenitor cells (Baker *et al*., 2011; Sousa-Victor *et al*., 2014). These evidence indicate that dysregulation of the Bmi-1/p16^Ink4a^ pathway likely plays important roles in provoking the aging-associated decline of stem or progenitor cell function and subsequent onset of degenerative diseases. However, because Bmi-1-KO mice die within 20 weeks after birth (van der Lugt *et al*., 1994), it is difficult to determine exactly where and when dysregulation of the Bmi-1/p16^Ink4a^ pathway occurs during aging *in vivo*.

To circumvent this problem, in the present study, we used *p16–luc* mice, in which *p16*^*Ink4a*^ expression can be monitored throughout the body using a bioluminescence imaging (BLI) technique (Yamakoshi *et al*., 2009). This approach, in conjunction with analysis of Bmi-1-KO mice and aged wild-type (WT) mice, uncovered a novel function of the Bmi-1/p16^Ink4a^ pathway in proliferation control of stem or progenitor cells in the submandibular glands (SMGs), which secrete saliva into the oral cavity (Young & van Lennep, 1978). Furthermore, our findings showed that the Bmi-1/p16^Ink4a^ pathway becomes dysregulated in SMGs during the aging process, which leads to induction of *p16*^*Ink4a*^ expression and the subsequent decline of SMG function. This unexpected role of the Bmi-1/p16^Ink4a^ pathway in the SMGs will likely provide new insights into the mechanism(s) underlying the aging-associated decline of SMG function and associated salivary gland hypofunction (SGH), which is a serious problem in the elderly population (Scott, 1977; Epstein *et al*., 1980; Pedersen *et al*., 1985; Yeh *et al*., 1998; Lenander-Lumikari & Loimaranta, 2000; Sreebny, 2000; van der Maarel-Wierink *et al*., 2013).

## Results

### Induction of *p16*^*Ink4a*^ expression in the SMGs of Bmi-1-KO mice

**Fig 1:**
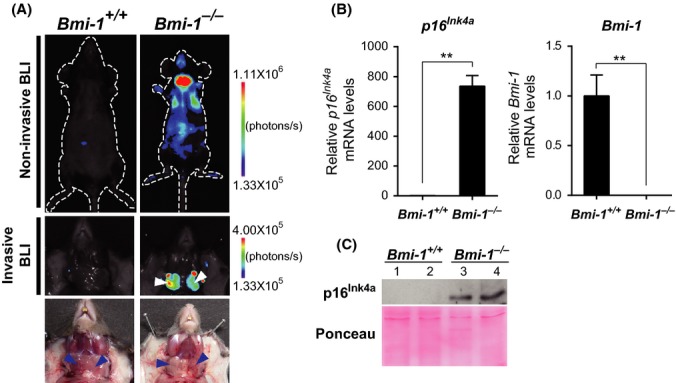
Visualization of increased *p16*^*Ink4a*^ expression in the SMGs of Bmi-1-KO mice (A) Twelve-week-old *p16–luc* mice lacking *Bmi-1* (*Bmi-1*^*−/−*^) or WT controls (*Bmi-1*^*+/+*^) were subjected to noninvasive BLI (top). The same mice were incised under anesthesia (middle). Representative images of five different experiments are shown. Mice were photographed in dimmed light (bottom). The color bar indicates photons with minimum and maximum threshold values. Arrowheads show the submandibular glands (SMGs). (B) qPCR was used to determine relative mRNA levels of *p16*^*Ink4a*^ and *Bmi-1* (left and right panels, respectively) in SMGs from *Bmi-1*^*+/+*^ (*n *=* *6) or *Bmi-1*^*−/−*^ (*n *=* *3) mice (10–15 weeks old). Results were standardized to *Gapdh* and expressed as the fold change of SMGs from *Bmi-1*^*−/−*^ mice compared with *Bmi-1*^*+/+*^ mice. Data are presented as means ±SD, **P <* *0.01. (C) A representative immunoblot of p16^Ink4a^ expressed by *Bmi-1*^*+/+*^ or *Bmi-1*^*−/−*^ mice. Ponceau staining of the membrane is shown as a loading control.

To unveil the physiological roles of Bmi-1 in the regulation of *p16*^*Ink4a*^ expression *in vivo*, we crossed *p16–luc* mice (*p16*^*Ink4a*^ reporter mice; Yamakoshi *et al*., 2009) onto a heterozygous *Bmi-1*-KO mice to produce *p16–luc* mice lacking *Bmi-1*. When these mice were analyzed using a noninvasive *in vivo* BLI technique, we observed a significant increase in signals throughout the body compared with control *p16–luc* mice, which was particularly enhanced in the cervical region (Fig.1A, top right). These mice were again subjected to invasive BLI under anesthesia, which identified SMGs as the main source of high-intensity bioluminescence (Fig.1A, middle and bottom right). Furthermore, endogenous *p16*^*Ink4a*^ mRNA and protein levels significantly increased in the SMGs of mice lacking *Bmi-1* (Fig.1B, C), indicating that *p16*^*Ink4a*^ expression is prematurely upregulated in SMGs in the absence of Bmi-1. Note that the expression level of *p19*^*Arf*^, another transcript produced by the *Ink4a/Arf* gene locus, was also increased in the SMGs of mice lacking *Bmi-1*, albeit to a lesser extent (Fig. S1A). However, because the expression level of *p21*^*Waf1/Cip1*^, a major downstream target of p19^Arf^, was almost unchanged (Fig. S1A), it is unlikely that *p19*^*Arf*^ plays a key role in SMGs in Bmi-1-KO mice. Moreover, the mRNA levels of other CDK inhibitors in SMGs remained largely unchanged by ablation of Bmi-1 (Fig. S1A). Together, these results suggest that regulation of *p16*^*Ink4a*^ gene expression by Bmi-1 may play key roles in SMG function in adult mice.

### Dysregulation of the Bmi-1/p16^Ink4a^ pathway in the aged SMG

**Fig 2:**
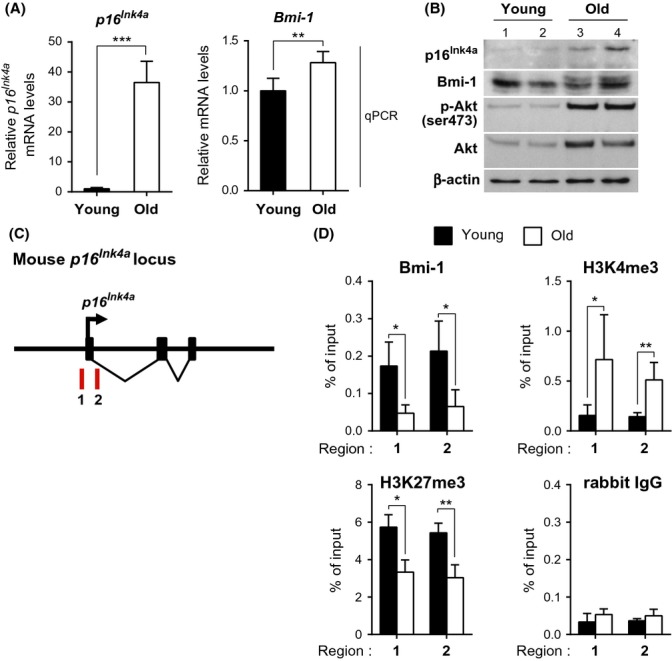
Increased *p16*^*Ink4a*^ expression and dysregulation of the Bmi-1/p16^Ink4a^ pathway in the SMGs of aged WT mice (A) Relative mRNA levels of *p16*^*Ink4a*^ and *Bmi-1* (left and right panels, respectively) in SMGs from young (8 weeks old) or old (24 months old) adult WT (WT) mice. Mean values of *p16*^*Ink4a*^ and *Bmi-1* mRNA levels in the young WT group were used as controls. Data are presented as mean ± SD, *n *=* *5, ***P <* *0.001; **P <* *0.01. (B) Representative immunoblot of SMG samples from young and old adult WT mice. β-actin was used as a loading control. (C) A schematic of the mouse *p16*^*Ink4a*^ locus. Amplified regions in (D) are shown as red bars. (D) Chromatin immunoprecipitation (ChIP) analysis using the indicated antibodies to detect binding of Bmi-1 to the *p16*^*Ink4a*^ locus in SMGs isolated from young (7 weeks old) or old (24–26 months old) adult WT mice. Data are presented as means ± SD, *n *=* *3–4, **P <* *0.01; *P <* *0.05.

Because p16^Ink4a^ expression levels increase in many different tissues during aging (Zindy *et al*., 1997; Krishnamurthy *et al*., 2004; Yamakoshi *et al*., 2009), we assumed that Bmi-1 likely regulates *p16*^*Ink4a*^ expression in SMGs. Therefore, we investigated the regulation of the *p16*^*Ink4a*^ locus during normal aging in WT mice. The SMGs of aged WT mice expressed significantly higher levels of *p16*^*Ink4a*^ than those of younger mice (Fig.2A, left and B). Notably, however, *Bmi-1* mRNA and protein levels in SMGs were slightly increased or remained unchanged (Fig.2A, right and B). However, Bmi-1 binding to the *p16*^*Ink4a*^ promoter region was significantly reduced in the SMGs of aged WT mice, which is consistent with the reduction in H3K27 me3 levels around the *p16*^*Ink4a*^ promoter region, as determined by chromatin immunoprecipitation (ChIP) analysis (Fig.2D). This coincided with the increase of H3K4 me3, an epigenetic mark of active chromatin around the *p16*^*Ink4a*^ promoter region (Fig.2D). In addition, it should be noted that the levels of phosphorylated (p)-AKT (Ak strain transforming) at Ser 473, a sign of AKT activation, which is known to phosphorylate Bmi-1 and inactivate its ability to bind the *p16*^*Ink4a*^ locus (Liu *et al*., 2012), were significantly increased in the SMGs of aged mice compared with those of young mice (Fig.2B). Collectively, these results indicate that although Bmi-1 mRNA and protein levels were unchanged, the activity of Bmi-1 was reduced, possibly through phosphorylation by AKT during the aging process *in vivo*. Consequently, the epigenetic silencing of *p16*^*Ink4a*^ by Bmi-1 is likely to be abolished in SMG parenchymal cells during aging. Similar to the results of Bmi-1-KO SMGs, the expression levels of other cell cycle inhibitors were unchanged during aging (Fig. S1B, C), thus supporting the idea that the Bmi-1/p16^Ink4a^ pathway plays key roles in controlling SMG function during aging.

### SMG function declines in Bmi-1-KO and aged WT mice

**Fig 3:**
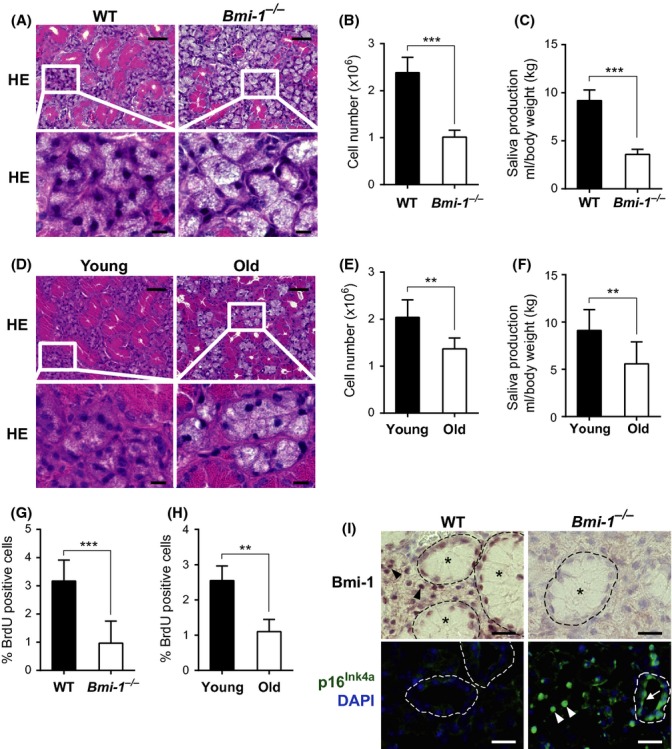
Submandibular abnormalities in Bmi-1-KO and aged WT mice (A) Hematoxylin and eosin (H&E) staining analysis of SMG sections showing sparse parenchymal cells in *Bmi-1*^*−/−*^ mice. Boxes denote regions shown below at higher magnification. Ages, WT, and *Bmi-1*^*−/−*^ (10 weeks old). Scale bars: 50 μm, upper panels; 10 μm, magnified views, lower panels. (B) Absolute cell numbers in SMGs from WT or *Bmi-1*^*−/−*^ mice. Data are presented as means ± SD, *n *=* *5, ***P <* *0.001. (C) Saliva secretion after an intraperitoneal injection of pilocarpine in WT or *Bmi-1*^*−/−*^ mice. Results were normalized to body weight (g). Data are presented as means ± SD, *n *=* *4, ***P <* *0.001. (D) H&E staining analysis of SMG sections showing sparse parenchymal cells in old adult WT mice. Ages, young (12 weeks old) and old (24 months old). Scale bars: 50 μm, upper panels; 10 μm, lower panels. (E) Absolute cell numbers in SMGs from young (10 weeks old) or old (20–24 months old) adult WT mice. Data are presented as means ± SD, *n *=* *7, **P <* *0.01. (F) Saliva secretion after intraperitoneal injection of pilocarpine in young (11–12 weeks old) or old (25–27 months old) adult WT mice. Results were normalized to body weight (g). Data are presented as means ± SD, *n *=* *10, **P <* *0.01. (G) Quantification of proliferating SMG cells in WT or *Bmi-1*^*−/−*^ (7–8 weeks old) mice as a percentage of BrdU-positive cells. Data are presented as means ± SD, *n *=* *6–7, ***P <* *0.001. (H) Quantification of proliferating SMG cells in young (8–12 weeks old) or old (22–24 months old) adult WT mice as a percentage of BrdU-positive cells. Data are presented as means ± SD, *n *=* *4, **P <* *0.01. (I) Immunohistochemical analysis of Bmi-1 (upper panels) and p16^Ink4a^ (green; lower panels) expression using SMGs from WT or *Bmi-1*^*−/−*^ mice. DNA was stained with 4′,6-diamidino-2-phenylindole (blue). Ductal structures are marked by dashed lines. Arrows and arrowheads show positive ductal and acinar cells, respectively. Asterisk, granular convoluted duct. Scale bars, 20 μm. [Correction added on 18 June 2015, after first online publication: The vertical axis unit in Figure 3C and 3F was previously incorrect and this has been amended in this version.]

To substantiate this idea, we performed morphometric and salivary secretion analyses using Bmi-1-KO mice or aged WT mice. A lower density of secretory parenchyma (Fig.3A) and a significant decrease in the number of cells (Fig.3B) were observed in the SMGs of Bmi-1-KO mice compared with those of WT littermates, indicating a loss of functional secretory parenchyma in SMGs lacking Bmi-1. Consistent with these findings, the volume of saliva produced by SMGs was substantially reduced in Bmi-1-KO mice, as shown by the results of pilocarpine stimulation tests (Fig.3C). Similar results were observed in aged WT mice compared with young WT mice (Fig.3D–F), suggesting that the loss of Bmi-1 function during aging possibly reduced the number of secretory parenchymal cells, resulting in an aging-associated decrease in saliva production.

Because Bmi-1 determines proliferative capacity, we tested whether the reduction in parenchymal cell number in the SMGs of Bmi-1-KO and aged WT mice was attributable to the decreased cell proliferation of SMG parenchymal cells. Bromodeoxyuridine (BrdU) incorporation analysis revealed that the percentage of proliferating SMG parenchymal cells was significantly reduced in Bmi-1-KO mice and aged WT mice (Fig.3G, H). Immunofluorescence staining analysis revealed that Bmi-1, but not p16^Ink4a^, was strongly expressed in the nuclei of acinar cells and ductal structures in young WT mice (Fig.3I, top). In contrast, expression levels of p16^Ink4a^, but not Bmi-1, were high in SMG acinar and ductal cells of Bmi-1-KO mice (Fig.3I, bottom). These results indicate that Bmi-1 may regulate *p16*^*Ink4a*^ expression in secretory parenchymal and ductal cells, and dysregulation of this regulatory pathway will lead to upregulation of *p16*^*Ink4a*^ expression, reduction in saliva-producing parenchymal cell proliferation and subsequent decrease in saliva production in Bmi-1-KO or aged WT mice.

### Putative SMG stem or progenitor cells require Bmi-1 function

To assess whether SMG stem or progenitor cells require Bmi-1, we employed an *in vitro* model to study SMG stem or progenitor cells that involve the isolation and culture of murine SMG cells as salispheres (Lombaert *et al*., 2008), which are derived from putative stem cells of ductal origin and comprise cells that express the stem cell markers Sca-1, c-Kit and Msi-1 (Lombaert *et al*., 2008). The salispheres formed from Bmi-1-KO mice expressed significantly higher levels of *p16*^*Ink4a*^ than those formed from WT littermates (Fig. S2B, left). Fewer salispheres were derived from Bmi-1-KO mice, and they incorporated significantly lower levels of BrdU and generated significantly fewer salispheres capable of self-renewal than those derived from WT mice (Fig. S2C–E). Moreover, *in vitro* differentiation analysis using a three-dimensional (3D) collagen matrix culture revealed that salispheres derived from Bmi-1-KO mice exhibited fewer and shorter branches on day 10 than those derived from WT littermates (Fig. S2F) and that the levels of *Muc19* and *Amy1* (Vreugdenhil *et al*., 1982; Das *et al*., 2009), which are markers of acinar cell differentiation, were reduced in Bmi-1-KO branched cells compared with those of WT littermates (Fig. S2G).

**Fig 4:**
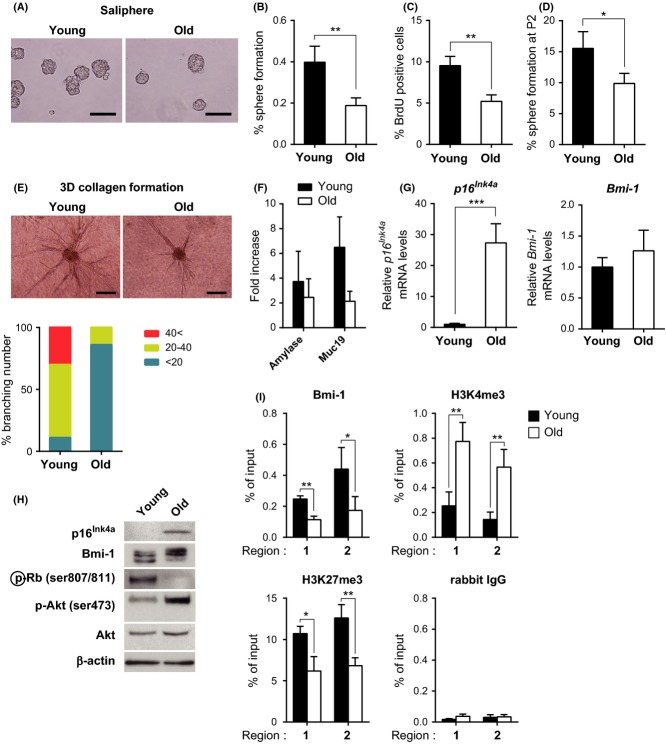
Aging-associated functional decline of SMG stem or progenitor cells. (A) Phase-contrast images of salispheres formed after 2 days in nonadherent cultures from young and old adult WT mice (left and right panels, respectively). Scale bars, 100 μm. (B) Effect of aging on the number of salispheres. Data are presented as means ± SD, *n *=* *5, **P <* *0.01. (C) Percentage of BrdU-positive cells in 10 representative salispheres from young (7–12 weeks old) or old (25 months old) adult WT mice. Data are presented as means ± SD, *n *=* *3, **P <* *0.01. (D) Percentage salisphere formation of CD24^hi^/CD29^hi^ cells at self-renewal passage-2 (P2) in enriched medium using from young (7–8 weeks old) or old (25–28 months old) adult WT mice. Data are presented as means ± SD of three independent experiments, *P <* *0.05. (E) Phase-contrast images of ductal-like branches on day 10 from young and old adult WT mice (left and right panels, respectively). Salispheres cultured for 2 days were transferred into a 3D collagen matrix. Scale bars, 200 μm. Histogram presenting the average branch numbers (%) of 25 cultured salispheres per mouse, *n *=* *4. (F) Fold increase of *Amy1* and *Muc19* mRNA levels from 2-day-old salispheres to ductal-like branches cultured for 18 days. Data are presented as means ± SD, *n *=* *3–4. (G) Relative *p16*^*Ink4a*^ and *Bmi-1* mRNA levels (left and right panels, respectively) in salispheres shown in (A) from young (8 weeks old) or old (24 months old) adult WT mice. qPCR results from salisphere RNA samples of individual mice in each group. Mean values of *p16*^*Ink4a*^ and *Bmi-1* mRNA levels in the young WT group were considered controls. Data are presented as means ± SD, *n *=* *4–5, ***P <* *0.001. (H) Representative immunoblot of salispheres from young and old adult WT mice for the indicated proteins. β-actin was used as a loading control. (I) ChIP analysis, using the indicated antibodies and salispheres shown in (A), of the *p16*^*Ink4a*^ locus. Data are presented as means ± SD of three independent experiments, **P <* *0.01; *P <* *0.05.

Furthermore, we determined whether aging affected the function of SMG stem or progenitor cells. In accordance with a previous report of reduced capability to grow secondary salispheres in aged WT mice (Feng *et al*., 2009), salispheres derived from aged WT mice were similar to those derived from Bmi-1-KO mice (Fig.4A–F). Taken together, these results indicate that Bmi-1 plays a role in the regulation of proliferation, self-renewal, and differentiation of putative SMG stem or progenitor cells. These results led us to speculate that the Bmi-1/p16^Ink4a^ pathway is associated with the functional decline of SMG stem or progenitor cells during the aging process.

### The Bmi-1/p16^Ink4a^ pathway is dysregulated in putative SMG stem or progenitor cells of aged WT mice

To test this idea, expression levels of *p16*^*Ink4a*^ and *Bmi-1*, binding of Bmi-1 and histone modifications around the *p16*^*Ink4a*^ promoter region were examined using salispheres from both young and aged mice. Indeed, the levels of *p16*^*Ink4a*^ expression were increased in salispheres from aged WT mice compared with those from young WT mice (Fig.4G, left and H). Note that although Bmi-1 mRNA and protein levels were not affected by aging (Fig.4G, right and H), Bmi-1 binding and the extent of H3K27 me3 modifications around the *p16*^*Ink4a*^ promoter region were substantially decreased (Fig.4I). In contrast, the extent of H3K4 me3 modifications around the *p16*^*Ink4a*^ promoter region was significantly increased in salispheres from aged mice (Fig.4I). Together, these results indicate that the Bmi-1/p16^Ink4a^ pathway is dysregulated through histone modifications in salispheres derived from putative SMG stem or progenitor cells during aging.

### Elevated p16^Ink4a^ levels inhibit proliferation of salispheres

To further examine the biological role of p16^Ink4a^ in SMGs, cells derived from the SMGs of young mice were transduced with one of two murine stem cell virus (MSCV) retroviral vectors expressing an internal ribosome entry site (IRES) and green fluorescent protein (GFP) with or without p16^Ink4a^ (pMSCV–IRES–GFP or pMSCV–p16^Ink4a^–IRES–GFP, respectively; Fig. S3A, B) and then subjected to BrdU incorporation analysis and 3D collagen matrix cultures. The proliferation rate of salisphere cultures overexpressing p16^Ink4a^ was substantially reduced (Fig. S3C), which is consistent with their smaller size (Fig. S3A, D) compared with those of control cells expressing GFP. Furthermore, ectopic p16^Ink4a^ expression in young WT SMG cells decreased the extent of branching (Fig. S3E) and levels of differentiation markers (Fig. S3F) of salispheres cultured for 7 or 17 days. Collectively, these results indicate that elevated levels of p16^Ink4a^ limit the proliferation and differentiation potential of putative adult SMG stem or progenitor cells. Thus, p16^Ink4a^ expression may inhibit the activities of SMG stem or progenitor cells.

### Ablation of *p16*^*Ink4a*^ partially rescues the abnormal phenotypes of Bmi-1-KO SMG

**Fig 5:**
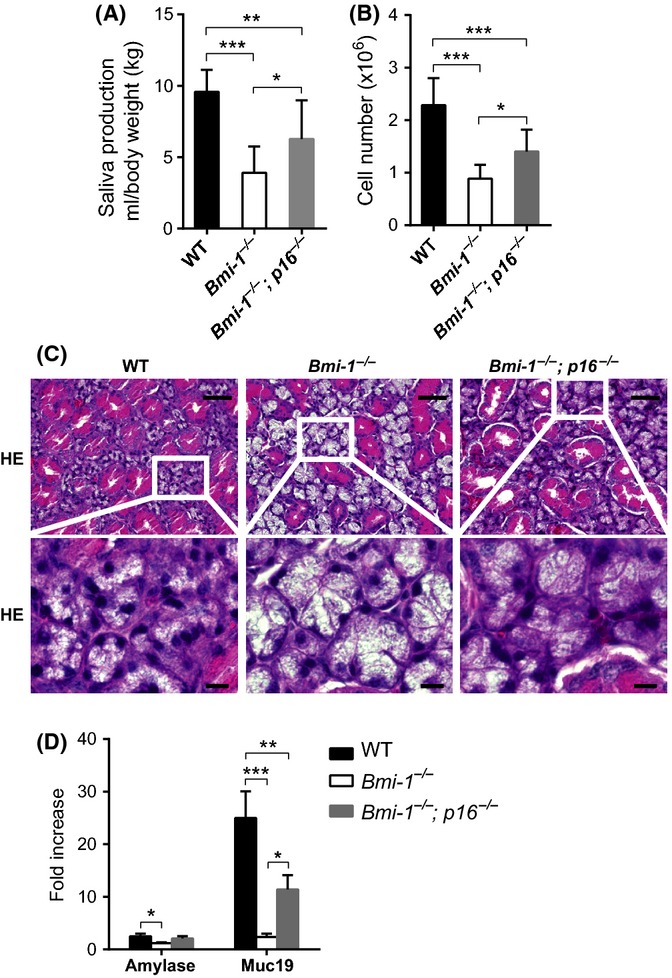
Deletion of *p16*^*Ink4a*^ partially rescues SMG abnormalities in Bmi-1-KO mice (A) Total volume of secreted saliva per gram of body weight after intraperitoneal injection of pilocarpine administered to each genotype (8–10 weeks old). Data are presented as means ± SD, *n *=* *9–11, ***P <* *0.001; **P <* *0.01; *P <* *0.05. (B) Absolute cell numbers of SMGs from WT or *Bmi-1*^*−*/*−*^ or *Bmi-1*^*−/−*^*; p16*^*−/−*^ mice (7–8 weeks old). Data are presented as means ± SD, *n *=* *7–10, ***P <* *0.001; *P <* *0.05. (C) H&E staining analysis of adult SMG sections from WT or *Bmi-1*^*−*/*−*^ or *Bmi-1*^*−/−*^*; p16*^*−/−*^ mice (10 weeks old). Scale bars, 50 μm (upper panels) and 10 μm (magnified views in lower panels). (D) Fold increase of *Amy1* and *Muc19* mRNA levels from 2-day-old salisphere to ductal-like branches cultured for 18 days. Data are presented as means ± SD, *n *=* *3–4, ***P <* *0.001; **P <* *0.01; *P <* *0.05. [Correction added on 18 June 2015, after first online publication: The vertical axis unit in Figure 5A was previously incorrect and this has been amended in this version.]

Finally, we asked whether the Bmi-1/p16^Ink4a^ pathway does play an important role(s) in the aging-associated decline of SMG function. The most straightforward approach to address this question would be to use *p16*^*Ink4a*^ KO mice. However, because *p16*^*Ink4a*^-KO mice die of cancer long before they reach the age at which most normal mice experience a decrease in SMG function, we asked whether *p16*^*Ink4a*^ deficiency can rescue the premature decrease in SMG function in Bmi-1-KO mice. To this end, we generated double-mutant mice lacking both *Bmi-1* and *p16*^*Ink4a*^ (Sharpless *et al*., 2001). Notably, although deletion of *p16*^*Ink4a*^ had no effect on SMG development in young WT mice, defective saliva production by Bmi-1-KO mice was partially rescued by ablation of *p16*^*Ink4a*^ (Fig.5A). This coincided with a substantial increase in the total number and density of SMG cells and the expression levels of differentiation-specific genes in branched cells (Fig.5B–D). Taken together, these results indicate that the Bmi-1/p16^Ink4a^ pathway possibly plays a key role in the aging-associated decline of SMG function, at least to some extent.

## Discussion

For many years, aging was believed to be an inevitable and random deterioration of the body, leading to loss of physiological function and increased vulnerability to disease and eventual death. Recent studies, however, reveal that the aging process, similar to other biological processes, is subject to control by various signaling pathways and gene expression patterns. Bmi-1, a PcG epigenetic regulator that blocks expression of the p16^Ink4a^ CDK inhibitor, plays a key role in aging, and dysregulation of the Bmi-1/p16^Ink4a^ pathway is believed to cause failure of adult stem cell homeostasis and the onset of aging-associated degenerative diseases (Dhawan *et al*., 2009; Sousa-Victor *et al*., 2014). However, because Bmi-1-KO mice die within 20 weeks after birth, it is difficult to determine exactly where and when dysregulation of the Bmi-1/p16^Ink4a^ pathway occurs during aging *in vivo*.

To circumvent this problem, we took advantage of using *p16–luc* mice, a recently developed transgenic mouse model that carries the entire human *p16*^*Ink4a*^ locus (Yamakoshi *et al*., 2009). Note that this human chromosome segment was engineered to express a fusion protein of human p16^Ink4a^ and firefly luciferase (p16–luc) without deleting any genomic DNA sequences of the *Ink4a/Arf* locus (Yamakoshi *et al*., 2009). This is very important because Bmi-1 binds not only to the promoter region but also to the intron region of the *p16*^*Ink4a*^ gene locus (Bracken *et al*., 2007; Kotake *et al*., 2007). Moreover, expression of the p16–luc fusion protein enables us to specifically measure *p16*^*Ink4a*^ expression, but not that of *Arf*, from this overlapping gene locus.

Using this approach together with Bmi-1-KO mice, we uncovered a novel function of the Bmi-1/p16^Ink4a^ pathway in the regulation of SMG function. Moreover, we found that this pathway was dysregulated during aging *in vivo*, leading to the induction of p16^Ink4a^ expression and subsequent decline of SMG stem or progenitor cell function and saliva production by the SMGs (Figs4). Although *p15*^*Ink4b*^ mRNA levels were increased to some extent in the SMGs of Bmi-1-KO mice and aged WT mice, the elevation in *p15*^*Ink4b*^ mRNA levels was much lower than that of *p16*^*Ink4a*^ (Fig. S1). Similar results were obtained in relation to p19^Arf^ (Fig. S1), although there was no evidence that the p53 pathway was involved in the aging-associated decline of SMG function (data not shown). These results indicate that the aging-associated decline of SMG function was attributable to dysregulation of the Bmi-1/p16^Ink4a^ pathway, at least to some extent. Because p16^Ink4a^-KO mice die of cancer long before they reach the age at which most normal mice experience a decline in SMG function, it is difficult to know whether dysregulation of the Bmi-1/p16^Ink4a^ pathway is responsible for the aging-associated decline of SMG function. However, because defects of saliva production in Bmi-1-KO mice were partially rescued by ablation of *p16*^*Ink4a*^ (Fig.5A), it is most likely that the Bmi-1/p16^Ink4a^ pathway plays a key role in the aging-associated decline of SMG function to some extent.

The obvious remaining question is whether or not our findings can be applied to humans. In *p16–luc* mice, we can successfully monitor the expression of human *p16*^*Ink4a*^ transcribed from the human *Ink4a/Arf* gene locus. As previously reported (Yamakoshi *et al*., 2009), human *p16*^*Ink4a*^ is expressed in the same way as mouse *p16*^*Ink4a*^ gene (Fig.1). These findings, together with the decrease (approximately 20%–30%) in parenchymal tissues (predominantly secretory acinar cells) in salivary glands over the adult lifespan of humans (Scott, 1977), strongly suggest that the Bmi-1/p16^Ink4a^ pathway may play an important role in human SMGs as well. Because poor oral hygiene is an important etiologic factor leading to aspiration pneumonia, which is a frequent cause of death in frail older people (van der Maarel-Wierink *et al*., 2013), further studies are warranted to elucidate the mechanism underlying the aging-associated functional decline of SMG and its associated SGH.

It is clear that these aging-associated degenerative diseases cannot only be explained by our finding of dysregulation of the Bmi-1/p16^Ink4a^ pathway because the loss of p16^Ink4a^ only partially rescued some of the anomalies in Bmi-1-KO SMGs. Thus, it is possible that other factors, such as alterations in receptor function and signal transduction (Olsen *et al*., 1997), are involved in the aging-related decline of SMG function. Nonetheless, our findings extend the current understanding of the molecular mechanisms underlying the aging-related decline of SMG function and associated degenerative diseases and will open up new possibilities for its control.

## Experimental procedures

### Animals, BLI and image acquisition

The *p16–luc* transgenic mice (C57BL/6), BLI technique and image acquisition methods were previously described (Yamakoshi *et al*., 2009). The *p16–luc* mice were crossed with *Bmi-1*^*+/−*^ mice (C57BL/6) (van der Lugt *et al*., 1994) to produce *p16–luc* mice lacking *Bmi-1*. Aged adult WT mice (C57BL/6) were purchased from the National Centre for Geriatrics and Gerontology Experimental Animal Facility (Obu, Aichi, Japan). Male animals were used for all experiments except for BLI.

### Determination of the volume of saliva

Mice were anesthetized and intraperitoneally injected with 1 mg kg^−1^ of pilocarpine (Nacalai Tesque, Inc., Kyoto, Japan), and 1 min later, saliva was collected from the mouth for 10 min using a micropipette (Ringcaps, Hirschmann Laborgeräte, GmbH & Co. KG, Eberstadt, Germany). The total volume of saliva was measured and calculated per body weight.

### Isolation of SMG cells

SMGs were dissected, and cells were isolated, counted and cultured as previously described (Lombaert *et al*., 2008) with some modifications. In brief, cell suspensions were prepared by first mechanically disrupting the glands followed by enzymatic digestion with collagenase type II, hyaluronidase and CaCl_2_ at 37 °C for 40 min and then with 25 U of dispase at 37 °C for 1 h. After filtering, primary cells were further filtered and suspended in Dulbecco’s modified Eagle’s medium/F12 medium (Invitrogen Corporation, Carlsbad, CA, USA) supplemented with N2, GlutaMAX™, 20 ng ml^−1^ of epidermal growth factor, 20 ng ml^−1^ of fibroblast growth factor-2 and 10 μg ml^−1^ of insulin, penicillin and streptomycin (salisphere medium).

### Cell culture

For cell differentiation assays, 2-day-old primary salispheres were cultured in collagen 3D matrix (Cellmatrix Type I-A; Nitta Gelatin, Inc., Osaka, Japan) for 8 or 18 additional days. The average number of branches in 25 cultured salispheres was determined, and the branches were then released from the gelled matrix by depolymerization using collagenase L (Nitta Gelatin, Inc.) and used for quantitative real-time polymerase chain reaction (qPCR) analysis.

The self-renewal of salispheres was performed as previously described (Nanduri *et al*., 2014). In brief, CD24^hi^/CD29^hi^ subsets were sorted from primary salispheres and cultured in Matrigel (BD Biosciences, San Jose, CA, USA) with enriched medium (salisphere medium + Rho-inhibitor, Y-27632) for 7 days to induce secondary salisphere formation. Secondary salispheres were passaged two times every 7 days, and self-renewal was evaluated as the percentage of cells capable of forming salispheres at passage 2.

For retroviral infection experiments, isolated SMG cells from WT 7- to 8-week-old mice were allowed to adhere to poly-D-lysine/laminin-coated dishes (BD Biosciences) in salisphere medium. After 24 h, viral supernatant was added to the cells, which were then cultured for an additional 24 h. The cells were harvested, the medium was replaced, and the cells were subjected to nonadherent culture to form salispheres for an additional 1 or 3 days before the differentiation or immunoblot assays, respectively. Images were acquired using an inverted microscope (IX71; Olympus Corp., Tokyo, Japan) equipped with an UPlanFL 10× objective using dp controller software.

### qPCR and ChIP

Primer sequences and the methodological details of qPCR and ChIP can be found in the Experimental Procedures of Supporting information.

### Histology, immunohistochemistry, and immunocytochemistry

Detailed descriptions of the histological, immunohistochemical, and immunocytochemical analyses can be found in the Experimental Procedures of Supporting information.

### Immunoblotting

Immunoblotting was performed using the following antibodies: rabbit anti-Bmi-1 (#5856; Cell Signaling Technology, Inc., Beverly, MA, USA), rabbit anti-p16 (sc1207; Santa Cruz Biotechnology, Inc., Santa Cruz, CA, USA), rat anti-p19Arf (sc32748; Santa Cruz Biotechnology, Inc.), rabbit anti-p15 (sc613; Santa Cruz Biotechnology, Inc.), rabbit anti-p18 (sc865; Santa Cruz Biotechnology, Inc.), rabbit anti-phospho-Rb (ser807/811) (#9308; Cell Signaling Technology, Inc.), rabbit anti-phospho-Akt (ser473) (#4060; Cell Signaling Technology, Inc.), rabbit anti-Akt (#4691; Cell Signaling Technology, Inc.), and mouse anti-β-actin (A5316; Sigma-Aldrich, St. Louis, MO, USA).

### Statistical analysis

Mice were randomly assigned to each group. The sample size (*n*) of each group is described in the corresponding figure legends. Results are presented as mean ± standard deviation (SD) of a number (*n*) of independent experiments. Statistical significance was determined using a two-tailed unpaired *t*-test or Welch’s *t*-test and one-way anova using graphpad prism software (http://www.graphpad.com/scientific-software/prism/).

## Supporting Information

**Fig. S1** Analysis of CDKI levels in the SMGs of WT or Bmi-1-KO, young or old adult WT mice. **Fig. S2** Salispheres require Bmi-1 for normal stem or progenitor cell activities. **Fig. S3** Elevated p16^Ink4a^ levels inhibit the proliferation of salispheres. **Appendix S1** Complete experimental procedures
