# Supplementary material for: Dysregulation of the Bmi-1/p16Ink4a pathway provokes an aging-associated decline of submandibular gland function
Source: Aging Cell. 2015 Mar 31;14(4):616–24. doi: 10.1111/acel.12337 (PMC4531075; doi:10.1111/acel.12337)
Supplement: Supplementary file 1 [file acel0014-0616-sd1.zip › SI Experimental Procedures.docx]

**Supporting Information**

**Experimental Procedures**

**Quantification of mRNA levels using qPCR**

Total RNA was isolated from mouse tissues or cultured cells using TRIzol Reagent (Invitrogen); contaminating genomic DNA was removed using the RNeasy Micro Kit (Qiagen, Venlo, Netherlands) and RNA was converted to cDNA using ReverTra Ace Reverse Transcription Reagents (Toyobo, Osaka, Japan). Relative mRNA levels were determined using the SYBR Green I detection chemistry system (Toyobo) with a Bio-Rad CFX96 Touch Real-Time PCR Detection system. Primer sequences used are as follows:

*Gapdh*: 5’-CAACTACATGGTCTACATGTTC-3’ (forward)

5’-CGCCAGTAGACTCCACGAC-3’ (reverse)

*p16^Ink4a^*: 5’-GAACTCTTTCGGTCGTACCC-3’ (forward)

5’-CGAATCTGCACCGTAGTTGA-3’ (reverse)

*p19^Arf^*: 5’-TGAGGCTAGAGAGGATCTTGAG-3’ (forward)

5’-CAGAAGAGCTGCTACGTGAA-3’ (reverse)

*p15^Ink4b^*: 5’-CCAACGCCCTGAACCGC-3’ (forward)

5’-AGGTCTGGTAAGGGTGGCA-3’ (reverse)

*p18^Ink4c^*: 5’-CAGATTAACCATCCCAGTCCTT-3’ (forward)

5’-CCCCTTTCCTTTGCTCCTAA-3’ (reverse)

*p19^Ink4d^*: 5’-AATGTGACCCAAGGCCACT-3’ (forward)

5’-TTTCCTCTTTTGTTGACAAGTAACC-3’ (reverse)

*p21^Waf1/Cip1^*: 5’-TGTCTTGCACTCTGGTGTCT-3’ (forward)

5’-TGAGGGCTAAGGCCGAAGAT-3’ (reverse)

*Bmi-1*: 5’-TTTTATGCAGCTCACCCGTC-3’ (forward)

5’-TCACCTCTTCCTTAGGCTTCTC-3’ (reverse)

*α-Amylase*: 5’-GGTGCAACAATGTTGGTGTC-3’ (forward)

5’-ACTGCTTTGTCCAGCTTGAG-3’ (reverse)

*Muc19*: 5’-GCAACCCCACAGGCTTAGTG-3’ (forward)

5’-TTTGAATCGTAGATTCTCTCTTCTTCTG-3’ (reverse)

**Histology and immunohistochemistry**

Mice were sacrificed, and submandibular glands (SMG) were immediately isolated, rinsed in phosphate-buffered saline (PBS) and fixed in 4% paraformaldehyde or embedded in Tissue-Tek OCT compound (Sakura Finetek, Tokyo, Japan) for preparing paraffin and frozen blocks, respectively. Paraffin-embedded SMG tissue was used for hematoxylin and eosin (H&E) and immunohistochemical analysis. Frozen tissue sections were processed for immunofluorescence analysis. Paraffin-embedded SMG tissue was cut into 4-μm sections, deparaffinized, rehydrated and boiled for 10 min in 1 mM EDTA for antigen retrieval. The slides were then washed in PBS, incubated in 1% hydrogen peroxidase, washed again and incubated with primary antibodies according to the manufacturer’s instructions after blocking for 10 min with Power Block solution (BioGenex, Fremont, CA, USA). Subsequently, the slides were washed in PBS and incubated with appropriate secondary antibodies and labelling dyes. Detection of peroxidase activity was performed using biotinylated anti-rabbit IgG (Vector Laboratories, Inc., Burlingame, CA, USA) followed by incubation with the Elite ABC Kit (Vector Laboratories) and liquid DAB (DAKO, Glostrup, Denmark), and counterstained with haematoxylin. The secondary antibody used for immunofluorescence analyses was Alexa Fluor 488-conjugated goat anti-mouse (Molecular Probes, Carlsbad, CA, USA), and nuclei were stained with DAPI (Dojindo, Kumamoto, Japan). Frozen tissue was cut into 6-μm sections, air dried, fixed in 4% paraformaldehyde, washed and blocked for 30 min in Image-iT FX Signal Enhancer solution (Invitrogen). The slides were incubated with anti-p16 (sc1207, Santa Cruz Biotechnology, Inc., Dallas, TX, USA) antibody. The secondary antibodies were AlexaFluor 488-conjugated goat anti-rabbit (Molecular Probes), and nuclei were stained with DAPI. After washing, tissue sections were mounted with ProLong Gold Antifade Reagent (Invitrogen).

The primary antibodies used were as follows: anti-Bmi-1 (#5856, Cell Signalling Technology, Inc., Beverly, MA, USA), anti-BrdU (555627, BD Pharmingen, Inc., San Diego, CA, USA) and anti-p16 (sc1207, Santa Cruz Biotechnology, Inc.). Images were acquired using a BIOREVO BZ-9000 (Keyence, Corp., Osaka, Japan) microscope equipped with a CFI Plan Apo λ 40x (972033, Nikon, Corp., Tokyo, Japan) objective with BZ-II software (Keyence, Corp.).

Morphometric measurement of BrdU incorporation was performed by intraperitoneally injecting 100 mg kg^−1^ body weight of BrdU (Sigma-Aldrich, Corp., St. Louis, MO, USA) into mice 24 h before they were killed. At least four mice per group were analyzed. Five sections separated by >200 μm were subjected to immunohistochemical analysis.

**Immunocytochemistry**

For cell proliferation studies, salispheres were pulsed with 10 μM BrdU (Sigma-Aldrich) for 6 h at 37°C, fixed in 4% paraformaldehyde for 10 min, embedded in Histogel (Richard-Allan Scientific, Waltham, MA, USA) and then processed in paraffin. Deparaffinised sections were stained with an antibody against BrdU (555627, BD Pharmingen, Inc.).

**Chromatin immunoprecipitation (ChIP)**

Discrete SMG cells or salispheres were fixed in 1% formaldehyde at room temperature for 10–15 min. The cross-linking reaction was quenched with 0.125 M glycine followed by two washes with cold phosphate-buffered saline. Pellets were re-suspended in 1% SDS lysis buffer, and chromatin was sonicated to generate DNA fragments. Solubilized chromatin was clarified by centrifugation at 12,000 ×*g* and the supernatant was incubated for 2 h with Protein A Mag Sepharose (GE Healthcare, Little Chalfont, UK) blocked with bovine serum albumin. Cleared chromatin was incubated with a rabbit antibody against anti-Bmi-1 (#5856; Cell Signaling Technology, Inc.), anti-H3K27 me3 (07-449; Millipore, Billerica, MA, USA), anti-H3K4 me3 (ab8580; Abcam, Cambridge, UK) and anti-IgG control (#3900; Cell Signaling Technology, Inc.) at 4°C overnight. Immune complexes were bound to pre-blocked Protein A Mag Sepharose at 4°C for an additional 2 h. The precipitates were thoroughly washed and extracted twice in the elution buffer. To remove cross-links, the DNA–protein complexes were incubated in 0.2 M NaCl at 65°C for 6 h followed by a 20-min incubation at 37°C with 0.05 mg mL^−1^ RNase A and a 1-h incubation at 55°C with 0.1 mg mL^−1^ Proteinase K. After phenol–chloroform extractions of the eluate, DNA was precipitated with 2.5 volumes of ethanol using 0.3 M sodium acetate and glycogen as carrier at −80°C for 30 min. Precipitated DNA was pelleted, washed once with 70% ethanol, dried, resuspended in TE and quantified using real-time qPCR. PCR primer sequences used are as follows:

*p16^Ink4a^* Ps4: 5’-CTGTTTCAACTCCCAGCTCTC-3’ (forward)

5’-GATGGAGCCCGGACTACAGAAG-3’ (reverse)

*p16^Ink4a^* Ps5: 5’-GAACTCTTTCGGTCGTACCCC-3’ (forward)

5’- CAAAAGTTACCCGACTGCAGATG-3’ (reverse)
