# Supplementary material for: Dysregulation of the Bmi-1/p16Ink4a pathway provokes an aging-associated decline of submandibular gland function
Source: Aging Cell. 2015 Mar 31;14(4):616–24. doi: 10.1111/acel.12337 (PMC4531075; doi:10.1111/acel.12337)
Supplement: Supplementary file 1 [file acel0014-0616-sd1.zip › Figure S1-S3.pdf]

Figure S1

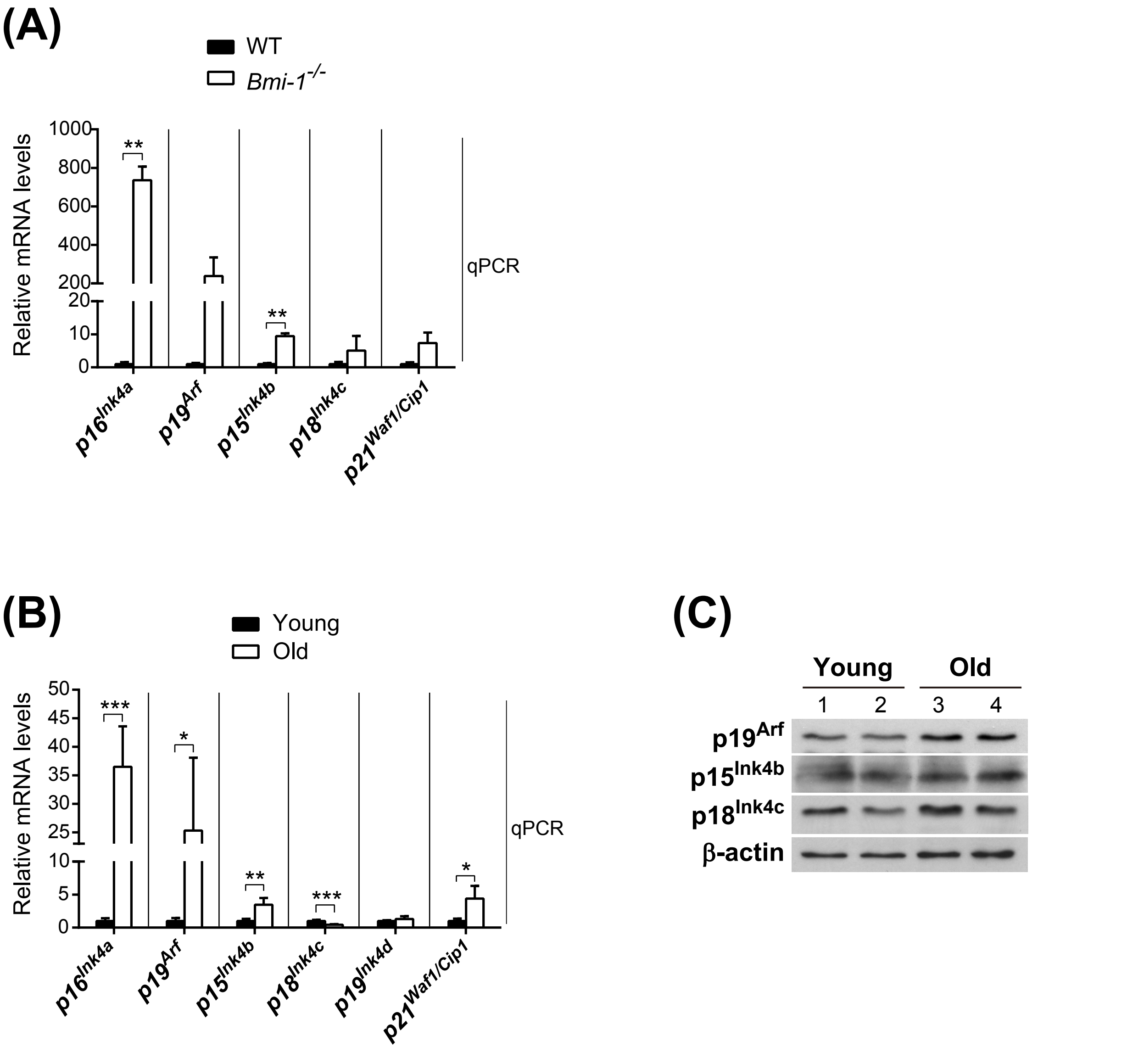

Figure S1 Analysis of CDKI levels in the SMGs of WT or Bmi-1-KO, young or old adult WT mice. (A) The levels of mRNAs encoding CDK inhibitors (CDKIs) of SMGs from WT (n = 6) or *Bmi-1*$^{-/-}$ (n = 3) mice (10–15 weeks old) were measured using RT-qPCR. Results are standardised to *Gapdh* and are expressed as the differences in expression detected in SMGs from *Bmi-1*$^{-/-}$ mice compared with those from WT mice. Data are represented as means ± s.d., **$P < 0.01$. (B) CDKI mRNA levels of SMGs from young (8 weeks old) or old (24 months old) adult WT mice were determined using RT-qPCR (n = 5 per group). The mean expression level of the young WT group was considered the control. Data are represented as mean ± s.d., ***$P < 0.001$; **$P < 0.01$; *$P < 0.05$. (C) Representative immunoblot of SMG proteins from young or old adult WT mice. β-actin was used as a loading control. β-actin is the same as that of Figure 2B.

Figure S2

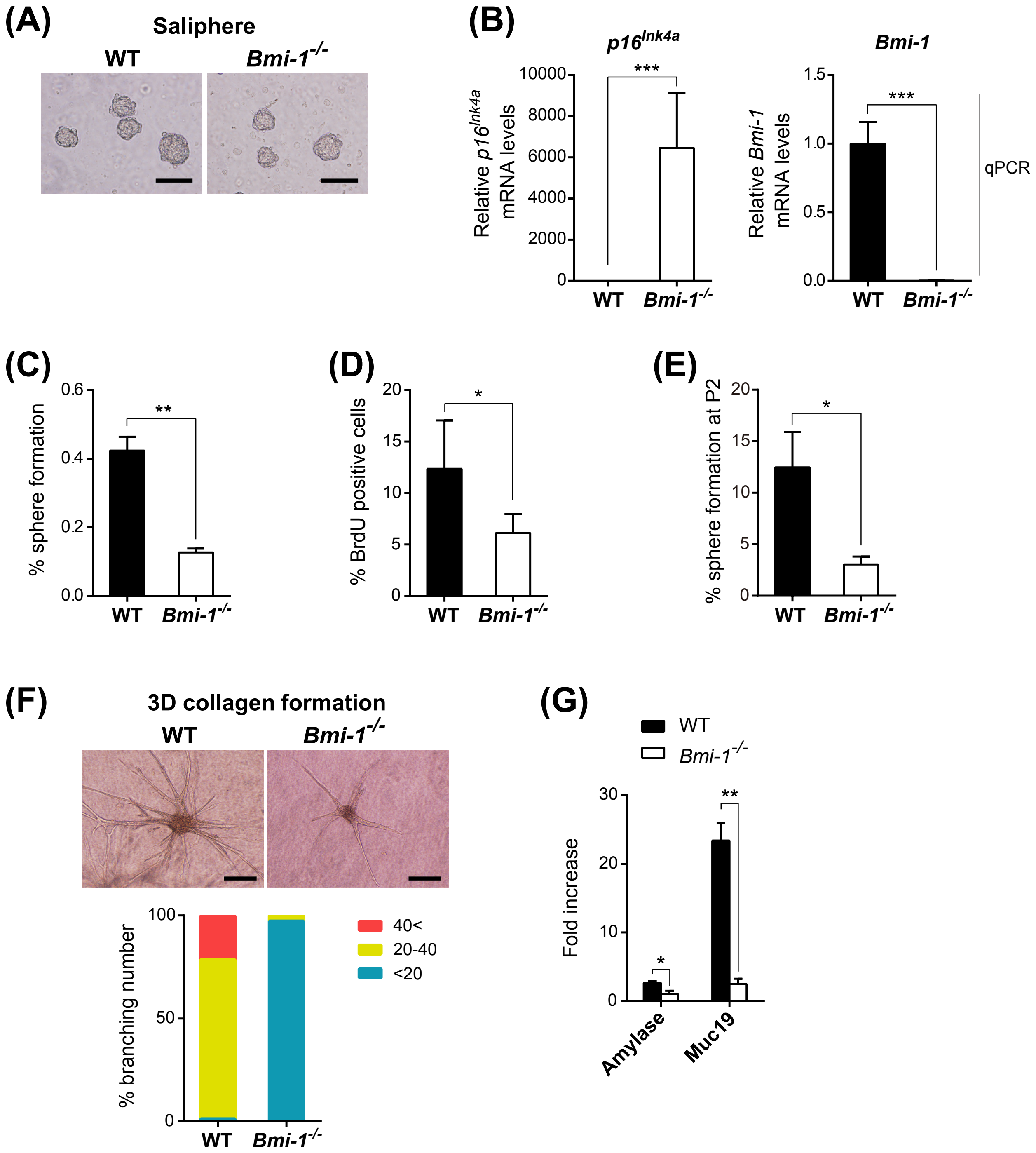

Figure S2 Salispheres require Bmi-1 for normal stem or progenitor cell activities. (A) Phase-contrast images of salispheres formed after 2 days in nonadherent cultures from WT or *Bmi-1*$^{-/-}$ mice (left and right panels, respectively). Scale bars, 100µm. (B) Relative *p16*^{Ink4a} and *Bmi-1* mRNA levels (left and right panels, respectively) in salispheres from WT or *Bmi-1*$^{-/-}$ (7–8 weeks old) mice. RT-qPCR results were acquired from salisphere RNA samples of individual mice of each group. Mean values of *p16*^{Ink4a} and *Bmi-1* mRNA levels in the WT group were considered as controls. Data are represented as mean ± s.d., n = 7, ***P< 0.001. (C) Effect of *Bmi-1* deficiency on the number of salispheres. Data are represented as mean ± s.d., n = 3, **P< 0.01. (D) Percentage of BrdU-positive cells in 10 salispheres from WT or *Bmi-1*$^{-/-}$ mice. Data are represented as mean ± s.d., n = 6, *P < 0.05. (E) Percentage of sphere formation of CD24^{hi}/CD29^{hi} cells at passage-2 (P2). With passage, Bmi-1-KO salisphere cultures undergo premature growth arrest compared with cultures from WT mice. Data are represented as mean ± s.d. of three experiments, *P < 0.05. (F) Phase-contrast images of ductal-like branches on day 10 from WT or *Bmi-1*$^{-/-}$ mice (left and right panels, respectively). Salispheres cultured for 2 days were transferred into a 3D collagen matrix. Scale bars, 200 µm. Histogram of the average number of branches (%) in 25 cultured salispheres per mouse, n = 3. (G) Fold increase of *Amy1* and *Muc19* mRNA levels from 2-day salispheres to ductal-like branches cultured for 18 days. Data are represented as mean ± s.d., n = 3, **P< 0.01; *P< 0.05.

Figure S3

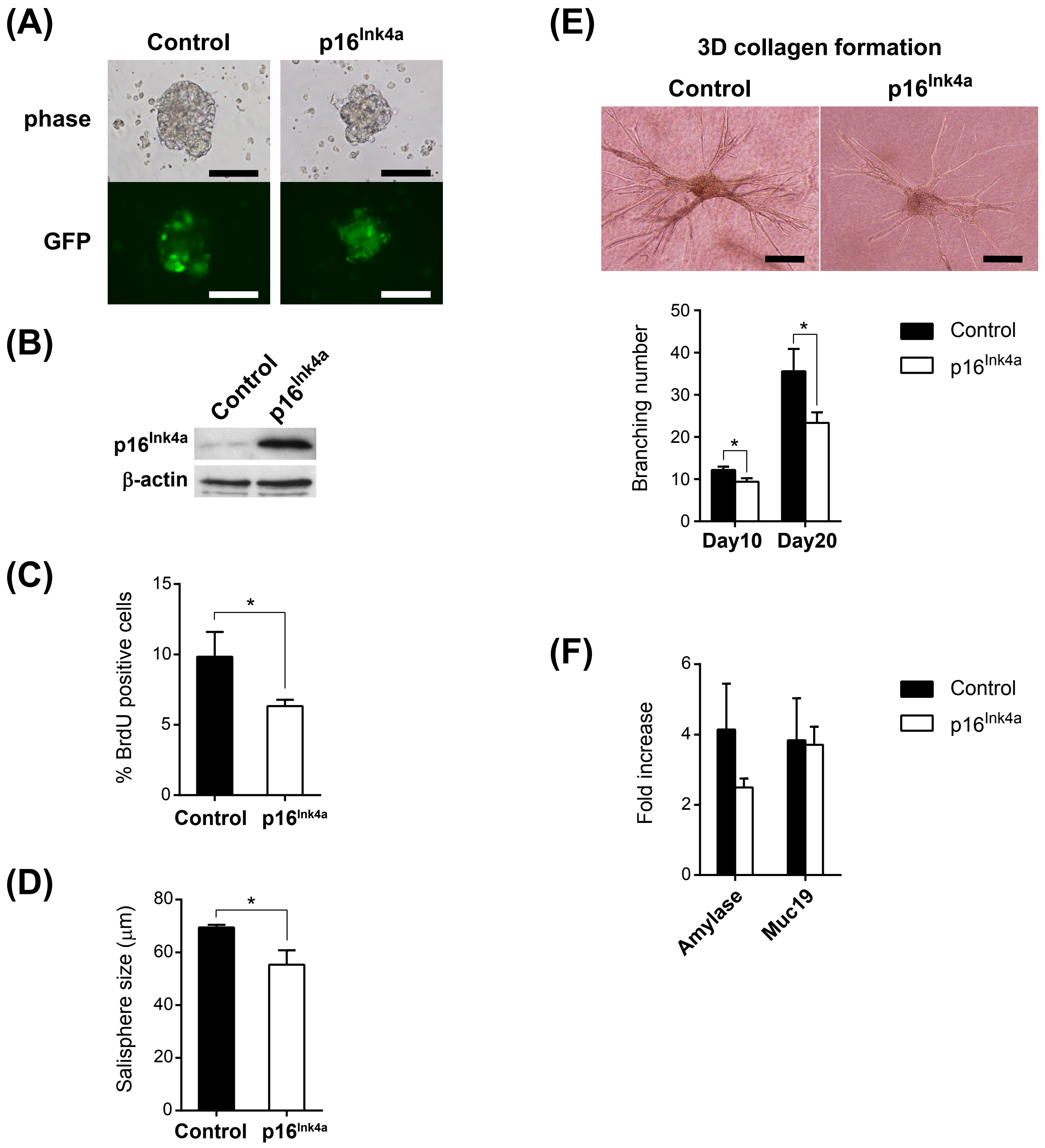

Figure S3 Elevated p16^{Ink4a} levels inhibit the proliferation of salispheres. (A) SMG cells were infected with retroviruses carrying an empty vector (EV) or construct encoding p16^{Ink4a}. Phase-contrast images (upper panels) and GFP images (lower panels) of the salispheres cultured for 5 days after viral infection. Scale bars, 100 μm. (B) Immunoblot analysis of p16^{Ink4a} expression in salispheres transduced with EV or p16^{Ink4a}. β-actin served as a loading control. (C) Percentage of BrdU-positive cells in salispheres infected with EV or p16^{Ink4a}. Values are presented as mean ± s.d. of three independent experiments, $*P < 0.05$. (D) The size of salispheres transduced with EV or p16^{Ink4a}. The diameters of 50 salispheres cultured for 3 days after viral infection were determined. The mean ± s.d. of three independent experiments is shown, $*P < 0.05$. (E) Typical images (upper panels) and the number (lower panels) of ductal-like branches that were generated from infected salispheres in (A). Scale bars, 200 μm. Data represent average numbers of branches in 25 cultured salispheres. The mean ± s.d. of three independent experiments are shown, $*P < 0.05$. (F) Fold increase of *Amy1* and *Muc19* mRNA levels from 3-day-old salispheres to ductal-like branches cultured for 17 days. The mean values ± s.d. of three independent experiments are shown.
